# Prognostic Significance of CD11b-, CD8-, and CD163-Positive Tumor-Infiltrating Immune Cells in Distal Bile Duct Cancer

**DOI:** 10.3390/jpm14101033

**Published:** 2024-09-27

**Authors:** Jae Hyung Choi, Joo Young Kim, Ki Rim Lee, Gyeong Yun Lee, Mineui Hong, Hye Won Hwang, Moo Yeol Lee, Mi Kyung Kim, Soon Auck Hong

**Affiliations:** 1 Department of Physiology, College of Medicine, Chung-Ang University, Seoul 06974, Republic of Korea; robbie23@naver.com; 2 Department of Pathology, College of Medicine, Chung-Ang University, Seoul 06974, Republic of Korea; jy0201@cau.ac.kr (J.Y.K.); gilim90@naver.com (K.R.L.); gyeongyooni@gmail.com (G.Y.L.); hongmineui@cau.ac.kr (M.H.); napinapi@cau.ac.kr (H.W.H.); mkkim@cau.ac.kr (M.K.K.)

**Keywords:** distal bile duct cancer, CD11b, CD8, CD163, tumor microenvironment, prognosis, immunotherapy

## Abstract

**Background**: Distal bile duct cancer is an aggressive malignancy. Tumor-infiltrating immune cells (TIICs) in the tumor microenvironment are crucial for predicting prognosis in various cancers. In this study, we analyzed TIICs based on CD11b, CD163, and CD8 expression, and evaluated their association with clinicopathologic factors and prognosis in distal bile duct cancer. **Methods:** A total of 90 patients who underwent curative resection for distal bile duct cancer were enrolled. We analyzed CD11b+ tumor-infiltrating myeloid cells (TIMs), CD163+ tumor-infiltrating macrophages (TAMs), and CD8+ tumor-infiltrating lymphocytes (TILs) using immunohistochemistry and tissue microarrays. The correlation between TIICs and clinicopathologic characteristics was assessed. **Results:** Low levels of CD11b+ TIMs (*p* < 0.001) and high levels of CD8+ TILs (*p* = 0.003) were significantly associated with improved overall survival (OS). A combined low level of CD11b+ TIMs and high level of CD8+ TILs was identified as an independent favorable prognostic factor (hazard ratio, 0.159; confidence interval, 0.061–0.410; *p* < 0.001). **Conclusions:** CD11b+ TIMs play a crucial role in the tumor microenvironment and the prognosis of distal bile duct cancer. The combined analysis of CD11b+ TIMs and CD8+ TILs can predict survival in patients with distal bile duct cancer.

## 1. Introduction

Distal bile duct cancer is a rare and aggressive malignant tumor, typically classified as an extrahepatic cholangiocarcinoma. It primarily originates from the biliary epithelium between the ampulla of Vater and the junction of the cystic duct [1,2]. The 5-year survival rate for patients who undergo surgical resection for distal bile duct cancers is only 35.4–44.4% [3,4]. While new targeted therapies based on genetic mutation, such as *FGFR2* and *IDH-1/2*, have shown promising results, they are predominantly applicable to intrahepatic cholangiocarcinoma rather than distal bile duct cancer [2,5]. Recently, *HER2*, *KRAS*, and *EGFR* mutations have been identified in some patients with distal bile duct cancer, making them potential candidates for targeted therapy. However, the overall benefits remain limited due to the small number of patients with these mutations [6]. As a result, there is an urgent need for new treatment options for distal bile duct cancer, with immunotherapy emerging as a promising approach.

Immunotherapy has achieved significant success as a treatment modality and has become a standard treatment for melanoma, lung cancer, genitourinary tract cancer, and head and neck cancer [7,8,9,10]. For immunotherapy to be effective, it is essential to understand the tumor microenvironment and the immune status of each tumor [11,12,13].

The tumor microenvironment consists of tumor cells themselves, along with various subsets of innate and adaptive immune cells, the vascular network, and stromal cells. These components interact through complex networks, involving growth factors, cytokines, chemokines, adhesion molecules, and extracellular matrices [14]. Among these, immune cells play dynamic roles in modulating either the promotion or suppression of tumor growth by interacting with other immune cells, and thus, they are now extensively utilized by immune therapy [15,16].

Immune dysregulation plays a crucial role in modulating the development and prognosis of distal bile duct cancer [17]; considerable research has been applied to elucidate the underlying mechanisms of immune dysregulation. However, only some studies can be used to study the tumor immune cells in distal bile duct cancer [18,19].

CD11b, the ligand that binds the subunits of the dimeric integrin CD11b/CD18b, is a receptor for both fibrinogen and endothelial intracellular adhesion molecule-1 and is expressed on most myeloid cells, including macrophages, monocytes, neutrophils, and some dendritic cell subsets. Recently, CD11b+ tumor-infiltrating myeloid cells (TIMs) were shown to interact with other immune cells, especially CD163+ tumor-associated macrophages (TAMs) and CD8+ tumor-infiltrating lymphocytes (TILs), in the tumor microenvironment [20,21]. Further, CD11b expression in myeloid cells can affect tumor characteristics and prognosis in various solid cancers, including gastric cancer, liver metastases from lung cancer, breast cancer, and intestinal tumors [22,23,24,25,26].

In this study, we aimed to analyze CD11b+ TIMs, CD8+ TILs, and CD163+ TAMs in relation to the clinicopathologic characteristics of distal bile duct cancer, and to assess the prognostic significance of the combined analysis of CD11b+ TIMs and CD8+ TILs.

## 2. Materials and Methods

### 2.1. Patients

This study was conducted as a non-randomized and retrospective analysis at a single institution. A total of 90 patients with distal bile duct cancer were enrolled in this study. All the participants were diagnosed with distal bile duct cancer and underwent curative resection between 2015 and 2020 at Chung-Ang University Hospital, Seoul, Republic of Korea. We subsequently collected cases with sufficient tissue samples for immunohistochemistry, along with the available clinicopathologic data. Clinical data, including patient age, sex, and overall survival (OS), were extracted from electronic medical records. The pathologic characteristics were meticulously re-evaluated, encompassing the depth of invasion, nodal status, lymphovascular invasion, perineural invasion, and resection margin status. The pathologic primary tumor (pT) and pathologic regional lymph node (pN) stages were reclassified according to the *TNM Classification of Malignant Tumours*, eighth edition.

### 2.2. Immunohistochemistry

Tissue microarrays (TMAs) were constructed by punch tissue cores (2 mm) from two representative areas and placed into recipient blocks. TMAs were processed with antigen retrieval and incubated primary antibodies against CD11b (1:200; Abcam, Cambridge, UK), CD8 (1:1000, C8/144B; Dako, Cambridge, UK), and CD163 (1:400; Abcam, Cambridge, UK). All the immunostains were carried out in the Venta Biotech automated system (Venta Medical System, Tucson, AZ, USA) according to the manufacturer’s protocol.

### 2.3. Analysis of Tumor-Infiltrating Immune Cells According to CD11b, CD8, and CD163 Expression

Immunostained slides were evaluated by two pancreaticobiliary pathologists (S.A.H and J.Y.K.) who were blinded to the clinicopathological information of the patients. Any discrepancies between the two pathologists were resolved by reviewing the cases together using a multi-head microscope until a consensus was reached.

The numbers of CD11b-, CD8-, and CD163-positive tumor-infiltrating immune cells (TIICs) were counted in five foci, with the highest density of immunostained cells found in the intratumoral area at a magnification of 400× (BX53; Olympus, Tokyo, Japan). High TIICs were defined as values above the medians for CD11b-, CD8-, and CD163-positive TIICs.

### 2.4. Statistical Analyses

Categorical variables were analyzed using the Chi-squared or Fisher’s exact test, as appropriate, while continuous variables were evaluated using Student’s T-test. The cut-off values for high or low levels of CD11b+ TIMs, CD8+ TILs, and CD163+ TAMs were determined using the receiver operating characteristic (ROC) curve and Youden’s index, with overall survival as the endpoint criterion. The correlation between the mean number of CD8+ TILs and CD163+ TAMs with CD11b+ TIMs was evaluated using a Pearson correlation test. OS was defined as the period from diagnosis to death from any cause, or the period from diagnosis to the last follow-up. The Kaplan–Meier method and log-rank test were used to plot survival times. Cox proportional hazards regression models determined the prognostic significance. The proportional hazards assumption was tested using the Schoenfeld residuals test. A two-sided *p*-value < 0.05 indicated statistical significance in all the tests and models. Data analyses were performed with the R statistical software version 4.3.2 (http://www.r-project.org, 25 July 2024).

## 3. Results

### 3.1. Clinicopathologic Characteristics

All the patients underwent curative resections, with R0 resections performed in 80 patients (88.9%) and R1 resections in 10 patients (11.1%). The median age was 68 years, and 53 patients (58.9%) were men. The median tumor size was 2.75 cm. According to TNM staging, 24 patients (26.7%) were pT1, 30 (33.3%) were pT2, and 36 (40.0%) were pT3. Regarding nodal status, 61 patients (67.8%) were N0, and 29 (32.2%) were N1/N2. Lymphovascular invasion and perineural invasion were observed in 45 patients (50.0%) and 71 patients (78.9%), respectively.

### 3.2. Correlation of CD11b+, CD8+, and CD163+ TIICs with Clinicopathologic Characteristics

**Figure 1:**
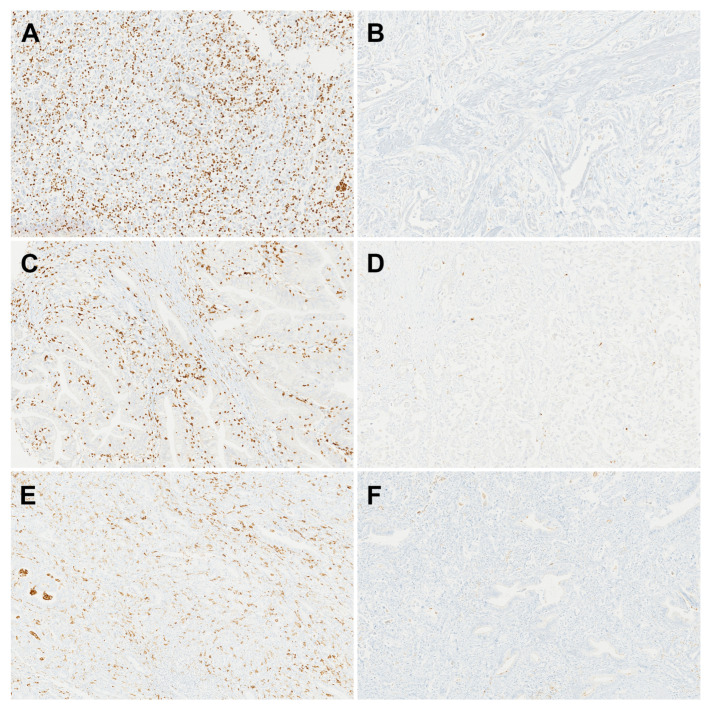
Representative immunohistochemistry of CD11b+ tumor-infiltrating myeloid cells (TIMs), CD8+ tumor-infiltrating lymphocytes (TILs), and CD163+ tumor-associated macrophages (TAMs). (**A**) High CD11b+ TIM level. (**B**) Low CD11b+ TIM level. (**C**) High CD8+ TIL level. (**D**) Low CD8+ TIL level. (**E**) High CD163+ TAM level. (**F**) Low CD163+ TAM level.

**Figure 2:**
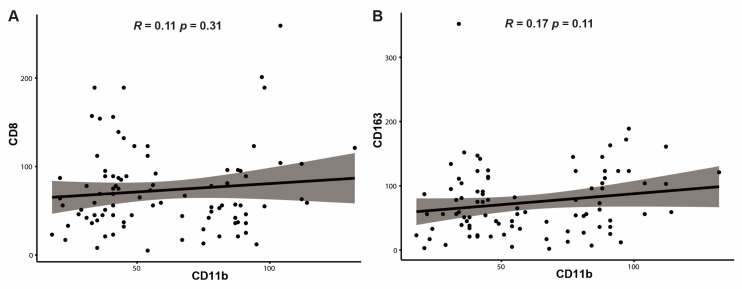
The correlation of the mean number of CD8+ tumor-infiltrating lymphocytes (TILs) and CD163+ tumor-associated macrophages (TAMs) according to those of CD11b+ tumor-infiltrating myeloid cells (TIMs) in distal bile duct cancer. The correlation is not identified significantly in CD8+ TILs (R = 0.11, *p* = 0.31) (**A**) and CD163+ TAMs (R = 0.17, *p* = 0.11) (**B**) with those of CD11b+ TIMs, respectively.

The median numbers of CD11b+ TIMs, CD8+ TILs, and CD163+ TAMs were 46, 58, and 67, respectively. Based on the results from the ROC curve and Youden’s index, the cut-off values were determined to be 58 for CD11b+ TIMs, 68 for CD8+ TILs, and 31 for CD163+ TAMs. Tumors were then classified as high or low based on these values. High-level CD11b+ TIMs were found in 38 patients (42.2%), high-level CD8+ TILs in 40 patients (44.4%), and high-level CD163+ TAMs in 70 patients (77.8%) (Table 1), (Figure 1A–F). The cases with low levels of CD8+ TILs were significantly associated with a high T stage (*p* = 0.002). The number of CD11b+ TIMs was not significantly correlated with the number of CD8+ TILs (R = 0.11; *p* = 0.311) (Figure 2A), or with the number of CD163+ TAMs (R = 0.17; *p* = 0.113) (Figure 2B).

### 3.3. Survival Effects with Respect to CD11b+, CD8+, and CD163+ TIICs

**Figure 3:**
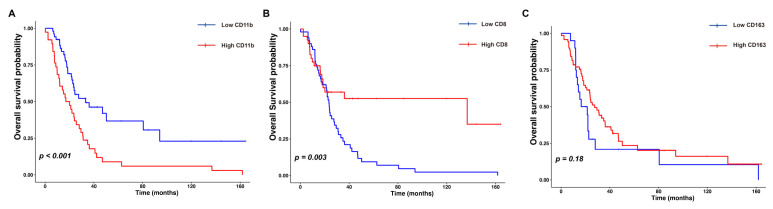
Prognostic analysis of CD11b+ tumor-infiltrating myeloid cells (TIMs), CD8+ tumor-infiltrating lymphocytes (TILs), and CD163+ tumor-associated macrophages (TAMs) in distal bile duct cancer. The Kaplan–Meier curves demonstrate a significant increase in overall survival (OS) in patients with low levels of CD11b+ TIMs (**A**) and high levels of CD8+ TILs (**B**); no significant difference in OS is observed according to CD163+ TMA levels (**C**).

The median OS was 22.25 months. The patients with either low levels of CD11b+ TIMs or high levels of CD8+ TILs had longer OS compared to those with high levels of CD11b+ TIMs or low levels of CD8+ TILs (low CD11b+, *p* < 0.001; high CD8+, *p* = 0.003). Conversely, CD163+ TAM status was not significantly related to OS (*p* = 0.18) (Figure 3A–C).

### 3.4. Clinicopathologic Features and Survival Effects with Respect to Concomitant Low CD11b+ and High CD8+ TIICs

**Figure 4:**
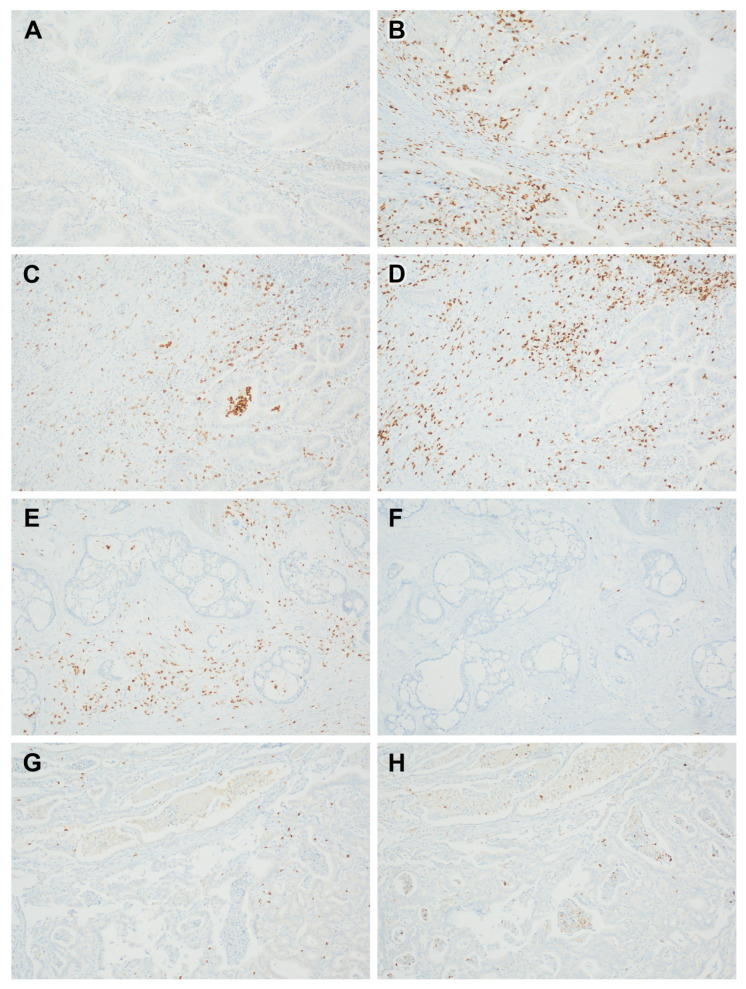
Representative immunohistochemistry for paired cases of CD11b+ tumor-infiltrating myeloid cells (TIMs) and CD8+ tumor-infiltrating lymphocytes (TILs): low CD11b+ TIMs (**A**) and high CD8+ TILs (**B**); high CD11b+ TIMs (**C**) and high CD8+ TILs (**D**); high CD11b+ TIMs (**E**) and low CD8+ TILs (**F**); and low CD11b+ TIMs (**G**) and low CD8+ TILs (**H**).

Concomitant low levels of CD11b+ TIMs and high levels of CD+8 TILs were found in 27 patients (30.0%) (Figure 4A–H). The tumors with low levels of CD11b+ TIMs and high levels of CD8+ TILs were significantly associated with a low T stage (*p* < 0.001) (Table 2).

**Figure 5:**
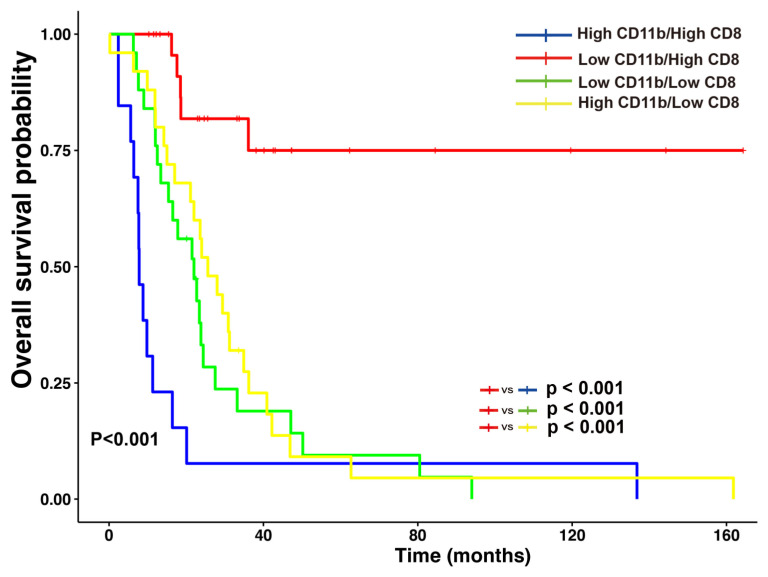
Combined analysis of CD11b+ tumor-infiltrating myeloid cells (TIMs) and CD8+ tumor-infiltrating lymphocytes (TILs) in distal bile duct cancer. Patients with a combined low level of CD11b+ TIMs and high level of CD8+ TILs have a favorable prognosis relative to other groups.

According to the status of combined CD11b+ TIMs and CD8+ TILs, patients had significantly increased OS (*p* < 0.001). In detail, the patients with combined low CD11b+ TIMs and high CD8+ TILs had longer OS than the patients with the following combinations: combined high CD11b+ TIMs and high CD8+ TILs (*p* < 0.001); combined low CD11b+ TIMs and low CD8+ TILs (*p* < 0.001); or combined high CD11b+ TIMs and low CD8+ TILs (*p* < 0.001) (Figure 5).

In a univariate Cox proportional hazards analysis, shorter OS was significantly and inversely correlated with high T stage, nodal metastasis, and R1 margin status, whereas the patients with combined low CD11b+ TIMs and high CD8+ TILs had significantly increased OS. A multivariate analysis revealed that combined low CD11b+ TIM with high CD8+ TIL status was an independent and significantly favorable prognostic factor (hazard ratio = 0.159; 95% confidence interval: 0.061–0.410, *p* < 0.001) (Table 3).

## 4. Discussion

In the present study, low levels of CD11b+ TIMs were associated with a favorable prognosis. The number of CD11b+ TIMs was not significantly associated with the numbers of CD8+ TILs and CD163+ TAMs. Further, a low level of CD11b+ TIMs combined with a high level of CD8+ TILs was an independent prognostic factor in distal bile duct cancer.

Tumor immunotherapy, including immune checkpoint inhibitors, vaccinations, immune cell therapies, and cytokine therapies, has become a practical strategy for tumor treatment [27,28,29]. For the feasibility of successful tumor immunotherapy, the characteristics of tumor immune status in each tumor must be evaluated.

CD11b expression has been identified in hematopoietic tumor cells and TIICs in various solid cancers [30,31,32]. Among TIICs, CD11b is predominantly expressed in myeloid cells, including dendritic cells, mononuclear cells, and myeloid-derived suppressor cells [33]. CD11b+ TIICs significantly contribute to the tumor microenvironment and play crucial roles in carcinogenesis. Targeting CD11b reduces myeloid cell infiltration, particularly in cells expressing S100A8 and matrix metalloproteinase-9, which are key factors in tumor regrowth [34]. In prostate cancer, tumor-derived cytokines drive CD11b+ cells to differentiate into osteoclasts, promoting bone metastasis [35]. Additionally, the BG34-200 ligand binds to a novel CD11b epitope, inducing monocyte-to-dendritic cell differentiation and enhancing T-cell activation, offering the potential for novel immunotherapies [36]. These mechanisms highlight the pivotal role of CD11b in both tumor progression and immune modulation.

In our study, the number of CD11b+ TIMs was similar to the numbers of CD8+ TILs and CD163+ TAMs. In previous studies, CD11b+ TIMs in gastric cancer and pancreatic cancer were abundant in tumors [24,37]. We confirmed that CD11b+ TIMs are one of the main components of TIICs in distal bile duct cancer.

Most studies suggest that CD163+ TAMs are associated with a poor prognosis in various solid cancers [38,39]. In our study, the level of CD163+ TAMs was not significantly associated with OS in distal bile duct cancer. Similarly, Miura et al. reported that CD163+ macrophages were not associated with OS [40], indicating that the prognostic role of CD163+ TMAs per se is limited in distal bile duct cancer.

CD11b can modulate some TIICs within the tumor microenvironment. Dendritic cells expressing major histocompatibility complex II^+^/CD11b^+^/CD11c^high^ can suppress CD8+ T-cell function in tumors [41]. Schmid et al. reported that the inhibition of CD11b in myeloid cells can lead to immune suppressive macrophage polarization by regulating M2-related cytokines [21]. Despite comparing the quantitative numbers of TIICs characterized by CD11b+, CD8+, and CD163+ markers, we did not observe significant changes in CD8+ TILs and CD163+ TAMs in line with the level of CD11b+ TIMs. Our results suggest that CD11b+ TIMs do not affect the quantitative levels of CD8+ TILs and CD163+ TAMs in distal bile duct cancer.

Some studies suggest that CD11b+ TIMs contribute to a poor prognosis in solid cancers, including gastric cancer, and are associated with recurrence after chemoradiotherapy in head and neck cancer [20,24]. We also found that the patients with high levels of CD11b+ TIMs had reduced OS (*p* = 0.042).

High levels of CD8+ TILs are a good prognostic marker in various solid cancers [42,43]. We also identified that the patients with high levels of CD8+ TILs had a favorable prognosis (*p* = 0.003). OS in the patients with low CD11b+ TIMs and high CD8+TILs was significantly different from that in the patients with high CD11b+ TIMs and high CD8+TILs (*p* < 0.001). Moreover, the patients with combined low CD11b+ TIMs and high CD8+ TILs showed the best OS compared to the patients in the other combined groups. Based on our data, we concluded that the CD8+ T-cell antitumor effect could be hindered by CD11b+ TIMs in distal bile duct cancer.

According to the possible mechanism by which CD11b+ TIM suppresses the tumoricidal effect of CD8+ TILs, CD11b+ myeloid-derived suppressor cells regulate the entry of activated CD8+ TILs into the tumor site. However, our results in distal bile duct cancer indicate that the levels of CD11b+ TIMs do not significantly affect the number of CD8+ TILs.

In line with our results, Yu et al. demonstrated that activated antigen-specific Fas^+^CD8^+^ T-cells undergo apoptosis following interaction with FasL^+^CD11b^+^F4/80^+^ monocyte-derived macrophages that contributed to immune deserts in a murine model of liver metastasis. Thus, CD11b+ TIMs may hinder antitumor immunity by suppressing CD8+ T-cells [44]. Furthermore, another potential mechanism involves CD11b+IL-4Rα+ monocytes. These cells, activated by IFN-γ from T cells, produce IL-13 and IFN-γ, suppressing antigen-activated CD8+ T cells [45]. This immunosuppressive action reduces immune surveillance and the effectiveness of immunotherapy, highlighting the need to target CD11b+ myeloid cells to restore T-cell function and improve treatment outcomes.

Unlike our results, Duong et al. reported that CD11b+ conventional dendritic cells activated by type 1 interferon promote antitumor CD8+ T-cell immunity [46]. Moreover, CD11b activation facilitates pro-inflammatory macrophage polarization by inducing the expression of microRNA *Let-7a*, demonstrated in mouse models of melanoma, breast, and lung cancer. In contrast, inhibiting CD11b reduces *Let-7a* levels and increases cMyc expression, driving immune suppressive macrophage polarization, enhancing vascular maturation, and promoting faster tumor growth [21]. These conflicting results could stem from the heterogeneity of CD11b+ myeloid cells because CD11b expression is observed in various inflammatory cells, including tumor-infiltrating monocytes, TAMs, granulocytes, and dendritic cells, and rarely, T-cells, B-cells, and NK and NK-T cells [37]. TIIC proportions in each tumor can vary according to the type of tumor [47]. We assume that the conflicting role of CD11b+ myeloid cells might originate from the differences in dominancy between tumor immune suppression or the antitumor immunity of CD11b+ myeloid cells.

The first limitation of our study is that we could not evaluate the sub-lineages of CD11b+ TIMs. As previously mentioned, various myeloid cells, including macrophages, monocytes, neutrophils, and some dendritic cells, even lymphoid cells, can express CD11b [37]. Further studies are needed to clarify the subpopulations of CD11b+ TIMs that play a significant prognostic role and to determine how to modulate T-cell immunity in distal bile duct cancer. A second limitation is that the study design is non-randomized and retrospective. This resulted in a relatively small sample size, which hindered the ability to perform power calculations for the appropriate number of patients. Consequently, the cut-off values for CD11b+ TIMs and CD8+ TILs in this study may not be definitive. Further large-scale, prospective validation studies are needed to establish optimized cut-off values. Despite our study limitation, we believe that evaluating levels of CD11b+ TIMs and CD8+ TILs is helpful in predicting prognosis in patients with distal bile duct cancer.

## 5. Conclusions

In this study, TIICs, including CD11b+ TIMs and CD8+ TILs, showed a significant prognostic role in distal bile duct cancer. Of note, we found that the combination of a low level of CD11b+ TIMs with a high level of CD8+ TILs was an independent prognostic factor in distal bile duct cancer; moreover, CD11b+ TIMs may hinder the antitumor activity of CD8+ T-cells. Overall, our data suggest that the control of CD11b+ TIMs could be one of the keys to the antitumor effect in distal bile duct cancer.

## Figures and Tables

**Table 1:** Association between CD11b, CD8, CD163, and clinicopathologic findings of distal bile duct cancer.

	CD11b			CD8			CD163		
Variable	High (n = 38) (%)	Low (n = 52) (%)	*p*-Value	High (n = 40) (%)	Low (n = 50) (%)	*p*-Value	High (n = 70) (%)	Low (n = 20) (%)	*p*-Value
Age (mean ± SD)	66.7 ± 10.4	68.0 ± 9.6	0.557 *	66.1 ± 9.4	68.5 ± 10.3	0.247 *	66.9± 10.0	69.4 ± 9.5	0.317 *
Gender			0.626			0.981			0.510
Male	24 (63.2)	29 (55.8)		23 (57.5)	30 (60.0)		43 (61.4)	10 (50.0)	
Female	14 (36.8)	23 (44.2)		17 (42.5)	20 (40.0)		27 (38.6)	10 (50.0)	
Tumor size (mean ± SD)	3.2± 1.5	3.0 ± 1.3	0.726 *	3.0 ± 1.3	3.2 ± 1.5	0.549 *	3.1 ± 1.3	3.1 ± 1.5	0.936 *
Histologic grade			0.358			0.707			0.206
Well	8 (21.1)	16 (30.8)		9 (22.5)	15(30.0)		16 (22.9)	8 (40.0)	
Moderate Poorly	23 (60.5) 7 (18.4)	31 (59.6) 5 (9.6)		25 (62.5) 6 (15.0)	29(58.0) 6 (12.0)		43 (61.4) 11 (15.7)	11 (55.0) 1 (5.0)	
T stage			0.318			0.002			0.372
T1	7 (18.4)	17 (32.7)		15 (37.5)	9 (18.0)		21 (30.0)	3 (15.0)	
T2	14 (36.8)	16 (30.8)		17(42.5)	13(26.0)		23 (32.9)	7 (35.0)	
T3	17 (44.7)	19 (36.5)		18(20.0)	28(56.0)		26 (37.1)	10 (50.0)	
N stage			0.723			0.338			0.678
N0	24 (63.2)	37 (71.2)		30(75.0)	31(62.0)		46 (65.7)	15 (75.0)	
N1 N2	12 (31.6) 2 (5.3)	13 (25.0) 2 (3.8)		8 (20.0) 2 (5.0)	17(34.0) 2(4.0%)		21 (30.0) 3 (4.3)	4 (20.0) 1 (5.0)	
Lymphovascular invasion			1			0.832			1
Yes	19 (50.0)	26 (50.0)		19(47.5)	26(52.0)		35 (50.0)	10 (50.0)	
No	19 (50.0)	26 (50.0)		21(52.5)	24 (48.0)		35 (50.0)	10 (50.0)	
Perineural invasion			0.803			0.583			0.654
Yes	29 (76.3)	42 (80.8)		30(75.0)	41 (82.0)		54 (77.1)	17 (85.0)	
No	9 (23.7)	10 (19.2)		10(25.0)	9 (18.0)		16 (22.9)	3 (15.0)	
Margin status of the bile duct			0.386			0.189			0.560
R0	32 (84.2)	48 (92.3)		38(95.0)	42(84.0)		61 (87.1)	19 (95.0)	
R1	6 (15.8)	4 (7.7)		2 (5.0)	8 (16.0)		9 (12.9)	1 (5.0)	

SD, standard deviation; *, *t*-test; R0, clear resection margin; R1, positive resection margin on microscopic examination.

**Table 2:** Association between high CD11b/high CD8 and clinicopathologic findings of distal bile duct cancer.

	Low CD11b/ High CD8		
Variable	Present (n = 27) (%)	Absent (n = 63) (%)	*p*-Value
Age (mean ± SD)	66.6 ± 9.6	67.8 ± 10.1	0.585
Gender			0.782
Male	15 (55.6)	38 (60.3)	
Female	12 (44.4)	25 (39.7)	
Tumor size (mean ± SD)	3.0 ± 1.2	3.1 ± 1.5	0.633
Histologic grade			0.902
Well	7 (25.9)	17 (27.0)	
Moderate Poorly	17 (63.0) 3 (11.1)	37 (58.7) 9 (14.3)	
T stage			<0.001
T1	13 (48.1)	11 (17.5)	
T2	11 (40.7)	19 (30.2)	
T3	3 (11.1)	33 (52.4)	
N stage			0.178
N0	22 (81.5)	39 (61.9)	
N1 N2	4 (14.8) 1 (3.7)	21 (33.3) 3 (4.8)	
Lymphovascular invasion			0.358
Yes	11 (40.7)	34 (54.0)	
No	16 (59.3)	29 (46.0)	
Perineural invasion			0.310
Yes	19 (70.4)	52 (82.5)	
No	8 (29.6)	11 (17.5)	
Margin status of the bile duct			0.067
R0	27 (100)	53 (84.1)	
R1	0 (0)	10 (15.9)	

SD, standard deviation; R0, clear resection margin; R1, positive resection margin on microscopic examination.

**Table 3:** Univariate and multivariate analyses of factors associated with overall survival of distal bile duct cancer.

Clinicopathologic Factors	Univariate Analysis			Multivariate Analysis		
	HR	95% CI	*p*-Value	HR	95% CI	*p*-Value
Age (years)	1.006	0.980–1.033	0.649			
Gender						
Male						
Female	0.971	0.590–1.599	0.909			
Tumor size	0.993	0.828–1.190	0.937			
Histologic grade						
Well						
Moderate	1.410	0.800–2.484	0.235			
Poorly	1.372	0.615–3.062	0.440			
pT stage						
pT1						
PT2	4.967	2.061–11.974	<0.001	5.221	1.989–13.704	<0.001
PT3	9.584	4.008–22.920	<0.001	6.785	2.646–17.397	<0.001
pN stage						
pN0						
pN1	1.760	1.019–3.040	0.043	1.267	0.721–2.226	0.411
pN2	4.565	1.334–15.615	0.016	2.711	0.782–9.390	0.116
Lymphovascular invasion						
No						
Yes	1.595	0.970–2.624	0.066			
Margin status of the bile duct						
R0						
R1	2.046	1.030–4.066	0.041	0.969	0.479–1.962	0.931
Perineural invasion						
No						
Yes	0.764	0.406–1.436	0.403			
Low CD11b/ high CD8						
No						
Yes	0.124	0.050–0.311	<0.001	0.159	0.061–0.410	<0.001

R0, clear resection margin; R1, positive resection margin on microscopic examination.

## Data Availability

The data used to support the findings of this study are available from the corresponding author upon request.

## Abbreviations

The following abbreviations are used in this manuscript:

TICC	Tumor-infiltrating immune cells
TIM	Tumor-infiltrating myeloid cell
TAM	Tumor-infiltrating macrophages
TIL	Tumor-infiltrating lymphocytes
OS	Overall survival
TMA	Tissue microarray
pT	The pathologic primary tumor stage
pN	The pathologic regional lymph node stage
